# Protocol for systematic review of evidence on the determinants and influence of early glycaemic control in childhood-onset type 1 diabetes

**DOI:** 10.1186/s13643-015-0146-8

**Published:** 2015-11-12

**Authors:** Veena Mazarello Paes, Dimitrios Charalampopoulos, Amal R. Khanolkar, David Taylor-Robinson, Russell Viner, Julie Edge, Terence Stephenson, Rakesh Amin

**Affiliations:** Institute of Child Health, University College London, London, UK; Institute of Public Health, University of Cambridge, Cambridge, UK; Institute of Environmental Medicine, Karolinska Institutet, Stockholm, Sweden; University of Liverpool, Liverpool, UK; Oxford Children’s Hospital, University of Oxford, Oxford, UK

## Abstract

**Background:**

Landmark studies in adult-onset type 1 diabetes (T1D) populations indicate that improved glycaemic control through use of intensive insulin therapy is strongly associated with reduced risk for the development of diabetes-related complications and mortality in later years. However, it is unclear whether these associations can be extrapolated to childhood-onset T1D, given the influence of other important biological and psychosocial determinants of glycaemic control, particularly during adolescence. The aims of the review are (1) to investigate the impact of early glycaemic control (within the first 2 years after diagnosis) on subsequent glycaemic trends and risk of complications during the life course of childhood-onset T1D and (2) to identify the predictors of early glycaemic control in children and young people (0–25 years).

**Methods:**

The methods used in this study are systematic identification, review and mapping of quantitative (intervention and observational) and qualitative literature; assessing the effect and predictors of early glycaemic control in T1D (diagnosed ≤18 years) on risk or prevalence of later complications. An iterated search strategy, with no language or period restrictions, was applied to identify studies from six electronic databases. This will be supplemented by hand-searching (reference lists and contacting authors of studies meeting the inclusion criteria). Studies assessing glycaemic control within the first 2 years of diagnosis in children (at baseline) will be quality-assessed against predefined criteria and mapped descriptively to future health outcomes. Extracted data will be analysed and synthesised using narrative and forest plots or harvest plots for quantitative evidence and thematic analyses for qualitative studies. To get a deeper understanding of the predictors of early glycaemic control in reducing complications in childhood and adult life, we will integrate qualitative and quantitative evidence using mixed methods or parallel synthesis approach.

**Discussion:**

These linked reviews will be the first to systematically investigate the effects of early glycaemic control and integrate both the quantitative and qualitative evidence on predictors of early glycaemic control in childhood-onset T1D in reducing future diabetes complications. This will help identify and map current research and inform development of effective future interventions.

**Systematic review registration:**

PROSPERO CRD42015024546

**Electronic supplementary material:**

The online version of this article (doi:10.1186/s13643-015-0146-8) contains supplementary material, which is available to authorized users.

## Background

Recent studies indicate a reduction of life expectancy by over a decade, in people diagnosed with type 1 diabetes (T1D) [1–3]. Given the duration of glycaemic exposure, the risk for diabetes-related vascular diseases is likely to be greater in childhood-onset compared to adult-onset T1D [3–6]. The burden of T1D greatly impacts upon the quality of life of children and their families [7, 8]. These poor outcomes have been highlighted by health policy advisers [9] and are of relevance given the increasing worldwide incidence of childhood T1D [10–14], and in those aged under 5 years, in whom both prognosis and burden of disease are likely to be even worse [15–18]. The strongest modifiable predictor of complications in T1D patients is glycaemic control, which is measured as haemoglobin A1_c_ levels (HbA1_c_) [19–22]. Use of intensive insulin therapy in young children is associated with better glycaemic control (i.e. lower HbA1_c_ levels) [23].

Results from a recent systematic review of 18 clinical trials (with a total of 2254 T1D participants and a mean follow-up duration across studies varying between 1 and 25 years) showed that targeting intensive glycaemic control (four or more insulin injections per day or insulin pump therapy) did not improve all-cause mortality, but it reduced the relative risk of the composite macrovascular outcomes (0.63; confidence interval (CI) 0.41 to 0.96; *P* = 0.03) and diabetic nephropathy (0.37; CI 0.27 to 0.50; *P* < 0.00001), when compared to management with conventional insulin regimens (i.e. three or less insulin injections per day) [24]. However, the review could not assess the effect of targeting intensive glycaemic control on patients younger than 18 years and no macrovascular or microvascular outcomes were reported in the four small trials (sample sizes between 14 and 34), three of which included newly diagnosed T1D patients under 18 years, probably due to the short follow-up period of up to 1.5 years [25–28].

An updated Cochrane review with 12 trials (with a mean follow-up duration across studies varying between 1 and 6.5 years) concluded that tight glycaemic control (compared to less intense treatment targets) was beneficial in younger patients (age not specified), at early stages of disease (retinopathy: RR 0.27 (95 % CI 0.18 to 0.42); *P* < 0.00001; nephropathy: RR 0.56 (95 % CI 0.46 to 0.68); *P* < 0.00001; neuropathy: RR 0.35 (95 % CI 0.23 to 0.53); *P* < 0.00001) [29]. However, this review contained just one trial in young children aged 6–15 years with diabetes duration of 1–2 years [30], and most of the other included trials were conducted in the 1980s, when use of insulin pump therapy was less widespread.

Also, evidence from landmark studies indicates that improved glycaemic control in T1D through use of intensive insulin therapy is strongly associated with reduced complications risk and mortality [19, 31, 32]. Results from the Diabetes Control and Complications Trial (DCCT), a multicenter, randomised controlled clinical trial of 1441 people (including 195 adolescents) with T1D (1983–1993), provided evidence that early intensive glycaemic control conferred a significant reduction in risk of microvascular and macrovascular complications compared to conventional treatment, an effect which continued in subsequent years even after equalisation of metabolic control [21, 31, 33, 34]. Importantly, reduced mortality was also observed with the use of early intensive versus conventional insulin therapy use (43 deaths versus 64 deaths respectively among 1429 participants; hazard ratio 0.67 (95 % CI 0.46–0.99)) after 27 years from entry into the trial [31]. All-cause mortality was significantly higher among those with higher mean HbA1_c_ levels (hazard ratio (HR) = 1.56 (95 % CI 1.35–1.81 per 10 % relative increase in HbA1_c_); *P* < 0.001) and with renal disease (HR = 8.51(95 % CI 4.45–16.27); *P* < 0.001) during the 27-years of follow-up [33, 35].

Other studies have also suggested the beneficial effects of early glycaemic control in preventing future complications [36, 37]. Therefore, it appears that maintaining lower HbA1_c_ levels in the early years after diagnosis may be beneficial in adults for reducing future risk of complications, regardless of subsequent glycaemic control over the course of diabetes duration. Diabetologists use the term “metabolic memory” for these observations. However, the DCCT contained only 195 adolescents (13 to 17 years of age at entry), with T1D duration of 38 ± 20 months at the start of the trial [38]. Hence, the effect of insulin intensification from or near to diagnosis has not been robustly assessed in childhood-onset T1D. It would be important to note that adult outcomes cannot be extrapolated to childhood due to important biological and psychosocial determinants such as puberty, insulin resistance and adolescent risk-taking behaviours that are less relevant in adults [39].

Furthermore, some reports indicate that very early HbA1_c_ level track with future glycemic control, i.e. low or high HbA1_c_ levels within the first few months of diagnosis associate with low or high HbA1_c_ levels in later years, an effect which can persist for almost a decade [31, 40–43]. These studies were from USA and Europe, but the age range, duration of T1D, treatment and follow-up varied across studies. Additionally, the use of intensive insulin therapy in early childhood T1D is increasing but is not universal [44]. Also, many well-documented population-based registries lack robust and comparable data on glycemic control [45].

Our review will investigate whether HbA1_c_ levels in the first 2 years following diagnosis of T1D in children predicts future risk of complications and will quantify the extent to which the level of glycaemia in the first 2 years of diabetes tracks in adulthood/with increasing diabetes duration. We will also investigate the predictors of early glycaemic control and how these may influence the paediatric TID management plan. Our analyses may highlight a need for a change in early care processes in children with T1D, by providing an argument for more intensive diabetes treatments than are currently undertaken.

## Methods/study design

We will undertake two linked systematic reviews answering two main research questions, namely:

1. Is higher early HbA1_c_ (within 2 years of T1D diagnosis) associated with later complications in child-onset T1D patients (childhood-onset T1D defined as onset ≤18 years)?
2. What factors at diagnosis or soon after are associated with higher early HbA1_c_?

We will follow the methods for conducting systematic reviews, as described by the Evidence for Policy and Practice Information (EPPI) and Co-ordinating Centre [46]. The review process will be in four phases. Phase 1 (completed): iterative scoping stage to define the research question, refine the search strategy and outline the inclusion/exclusion and quality assessment criteria. This was followed by identification of literature by searching of electronic databases. Phase 2: descriptive mapping and synthesis of existing evidence by number, types and quality attributes of research studies on the topic. Phase 3: detailed data extraction and in-depth synthesis of quantitative studies [47]. Phase 4: thematic analysis of qualitative literature [48], followed by integration of these findings with the quantitative evidence by using parallel synthesis or mixed method approach [49]. This methodology of using qualitative research to explain quantitative evidence will provide a deeper understanding of the predictors influencing early glycaemic control in children and young people.

### Search strategy

After a number of initial iterative scoping searches, with input from experts in the field, the search strategy was refined to maximise sensitivity and specificity in capturing key publications. Three sets of search terms were used (see search strategy in Table 1) relating to population (children and young people diagnosed with TID), exposure (terms to capture observational, intervention, qualitative studies and review articles relating to early diabetes control) and outcome (complications, mortality or metabolic memory).

**Table 1:** Search strategy for research questions: Does early glycaemic control (intervention within two years post diagnosis) in childhood-onset T1D have an impact on subsequent risk of complications in childhood and adulthood? What are the predictors of early glycaemic control?

Population	Exposure	Outcome
Childhood or paediatric onset diabetes or juvenile diabetes diagnosis or newly diagnosed children or young persons or young people or children or young or adolescent or teen or youth or adult T1D patient or type 1 diabetes or T1D or type 1 diabetes mellitus or T1DM or DM1 or type 1 or IDDM or insulin dependent or non-insulin dependent or childhood onset diabetes or childhood onset T1D or auto-immune or autoimmune or sudden onset or uncontrolled or labile or brittle	Early diabetes control or HbA1c trajectories or HbA1c trends or glycaemic trajectories or glycosylated or HbA1c or A1c or Hemoglobin A or HbA(1c) level or glycaemic control or glucose control or diabetes control or early intensive intervention or intensive or conventional or standard or regular or optimised or tight control or strict control or usual or routine or therapy or treatment or intervention or management or insulin use or injection or dose insulin injections or intensive therapy or insulin pump	Diabetic or diabetes complications or complications or side effects or adverse events or acute complications or chronic complications or glycaemia or hyper glycaemia or hypo glycaemia or ketosis or diabetic ketoacidosis or DKA or nonketotic hyperosmolar coma or insulin resistance or autoimmune disease or urine albumin or urine albumin creatinine ratio or urine albumin excretion or microalbuminuria or macroalbuminuria or renal disease or diabetic nephropathy or nephropathy or dialysis or foot ulcer or amputation or retinopathy or blindness or vascular disease or vascular complications or microvascular disease or microvascular complications or macrovascular disease or macrovascular complications or cardiovascular disease or MI or myocardial infarction or stroke or coronary artery disease or cerebrovascular disease or peripheral vascular disease or blood pressure or BP or statin or death or mortality or Pathology or metabolism or metabolic memory

**Fig. 1:**
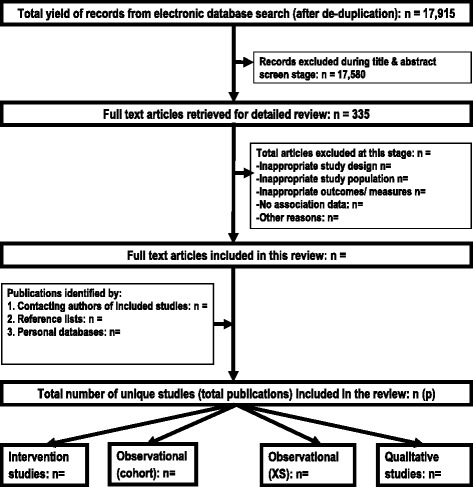
Flowchart presenting an overview of the search results

Six electronic databases (Medline and Embase (via OVID), Web of Science (via Thomson Reuters), Cinhal (via EBSCO), Scopus (via Elsevier) and Cochrane Library) were double-searched in parallel (by VMP and HC to minimise study selection bias) in December 2014, without time period or language restrictions, by using a combination of free text and Thesaurus or Mesh terms (see Additional file 1: Electronic database search strategy.pdf). All identified articles from individual databases (Medline, *n* = 13,039; Embase, *n* = 645; Web of Science, *n* = 2323; Cinahl, *n* = 984; Scopus, *n* = 1540 and Cochrane, *n* = 3242) were imported into an endnote file and de-duplicated, which resulted in 17,915 articles for further review. This will be supplemented by hand-searching reference lists and contacting authors of included studies and relevant reviews (see Fig. 1 for a flow diagram of the study selection process).

### Inclusion and exclusion criteria

Interventional studies, i.e. randomised control trials (RCTs) and non-RCTs, targeting glycaemic control (within 2 years of diagnosis of T1D in children and young people) and describing an association with health outcomes will be included. Observational, i.e. cohort and cross-sectional studies that quantified the association between early glycaemic control (within 2 years of diagnosis of T1D) AND risk of future complications in children and young people aged 0 to 25 years at baseline, will be included. Qualitative studies that give a deeper background understanding on the predictors of early HbA1_c_ in this age group will also be included.

Our exclusion criteria are as follows: non-human or animal studies, studies with population selected for other diseases/co-morbidities or clinical conditions, studies in adults aged more than 25 years at baseline, studies in other types of diabetes such as type 2 diabetes (T2D) or gestational diabetes. Quantitative studies not reporting clinical outcomes or quantitative studies that measured glycaemic control but did not describe an association with outcome variables will also be excluded. The overlapping eligibility criteria for the reviews are presented as inclusion/exclusion criteria in Table 2.

**Table 2:** Inclusion and exclusion criteria for review of evidence on the following: 1. Does early HbA1c predict later complications? 2. What factors predict early HbA1c?

Inclusion criteria	Exclusion criteria
For reviews 1 and 2	
Interventional studies (RCT’s and non-RCT’s) targeting glycaemic control (within 2 years of diagnosis of T1D) and described an association with health outcomes	Non-human studies
	Selection of population based on other diseases/co-morbidities
	Adults aged more than 25 years at baseline
	Studies on T2D
	Quantitative studies not reporting clinical outcomes
	Quantitative studies that measured glycaemic control but did not describe an association with outcome variables
Non-intervention/observational, i.e. cohort and cross-sectional (XS) studies that quantified the association between early glycaemic control (within 2 years of diagnosis of T1D) AND risk of future complications in children and young people aged 0 to 25 years at baseline	
For review 1: longitudinal studies with a follow-up of ≥5 years from diagnosis	
For review 2: qualitative studies that give a deeper background understanding on the topic	

### Study selection procedure

A 10 % proportion (1792) of the total abstracts and titles will be randomly selected and double screened (DC and VMP), based on a piloted screening protocol. Results will be compared to ensure less than 5 % discrepancy between reviewers. Any disagreements will be resolved through discussion and re-examining of abstract. Following which, all 17,915 abstracts and titles will be screened independently (VMP). Full texts of abstracts appearing to meet the inclusion criteria will be ordered for further review and data extraction. Descriptive mapping of existing evidence will be undertaken to establish gaps in evidence-base and to ascertain that there is sufficient data meriting review.

### Data extraction and quality assessment

A data extraction Excel spread sheet will be piloted to ensure consistency of data extraction between reviewers. One of the reviewers (VMP) will then systematically review and extract detailed data of all studies meeting the inclusion criteria. A proportion of the studies will be double-reviewed by a second reviewer, and any differences will be discussed and resolved. Details of data will be extracted according to study designs, i.e. interventional, observational and qualitative (see Table 3).

**Table 3:** Details of data extracted for different study designs

Intervention studies	Observational/non-intervention studies (cross-sectional and prospective)	Qualitative studies
Study id	Study id	Study id
Author	Author	Author
Year	Year	Year
Country	Country	Country
Age range	Age range	Age
Average age	Average age	Design
Sex (male to female ratio)	Sex (male to female ratio)	Number of participants
Design: cRCT, RCT, quasiRCT, before-after, etc.	Ethnicity	Sample/recruitment
Number of participants	Socioeconomic status	Findings/direct quotes
Sample/recruitment, e.g. general population representative sample or specialist groups (deprivation, ethnicity, geography, etc.)	Design (cross-sectional/prospective)	Author conclusions
Intervention details (pump/injection, duration, dosage)	Number of participants	Comments
Control	Sample/recruitment, e.g. general population representative sample or specialist groups	Author email
Setting (home, primary care, secondary care)	Exposure examined	
Intervention provider	Measurement of exposure	
Number of sites	Measurement conducted by Level of glycemic control	
Outcome (complications, metabolic memory—separate row for each outcome investigated)	Setting (home, primary care, secondary care)	
Measurement of outcome (objective)	Outcome (complications, metabolic memory—separate row for each outcome investigated)	
Analysis	Measurement of outcome (objective)	
Effect—point estimate	Analysis	
Effect—upper confidence interval		
Effect—lower confidence interval		
Follow-up duration	Effect	
Comments	Author email	
Author email	Comments	

Included studies will be systematically quality-assessed against pre-set quality criteria by using standard quality assessment checklists designed by the EPPI centre for specific (intervention, observational and qualitative) study designs (see Table 4).

**Table 4:** Quality assessment criteria by study design

For intervention studies	For observational (prospective cohort and cross-sectional) studies	For qualitative studies
Total quality assessment score (maximum of 8) was derived for the fulfilment of following criteria:	Total quality assessment score (maximum of 6) was derived for the fulfilment of following criteria:	Total quality assessment score (maximum of 12) was derived for the fulfilment of following criteria:
1) Randomisation	1) More than 50 participants analysed	1) Research questions clearly stated
2) Effect of intervention reported for all outcomes	2) Studies representing general population	2) Approach appropriate for the research question
3) Pre-intervention data on all outcomes	3) Prospective study design	3) Qualitative approach clearly justified
4) Post-intervention data on all outcomes	4) Adjusted/multivariate analysis	4) Study context clearly described
5) Allocation concealment	5) Objective measure of outcome	5) Role of the researcher clearly described
6) Blinding	6) Objective measure of exposure	6) Sampling method clearly described
7) Objective measurement of outcome		7) Sampling strategy appropriate for the research question
8) Retention >70 %		8) Method of data collection clearly described
Studies with small sample size (*n* < 50) and no control group were considered to provide lower quality evidence and not scored		9) Data collection method appropriate
		10) Method of analysis clearly described
		11) Analysis appropriate for the research question
		12) Conclusions supported by sufficient evidence

### Data syntheses

Extracted data will be analysed and synthesised using a narrative and either forest plots or harvest plots (for quantitative evidence) and thematic analyses (for qualitative studies). To get a deeper understanding of the predictors of early glycaemic control in reducing complications in childhood and adult life, we will integrate qualitative and quantitative evidence using mixed methods or parallel synthesis approach.

#### Intervention studies

Meta analyses will be attempted to synthesise data from RCTs and Non-RCTs, assessed with low risk of bias and the results presented as forest plots [50]. Subgroup analyses by intervention type will be undertaken, subject to data type and quality.

#### Observational studies

Harvest plots [51] will be used to summarise data if meta-analysis/meta-regression is not possible. The evidence will be presented as bar charts and symbols. Colour (black, dark grey and light grey) of bar will represent quality of study, with lighter bars representing studies of low quality. Height of bar will indicate the study size, and position of the bar will summarise the direction and strength of the association (++, +, 0, −, −−). Categorical and continuous outcome variable results will be consistently recoded, such that a single or double + symbolises higher risk for complications, and a single or double − symbolises a lower risk for complications [52, 53].

#### Qualitative studies

Evidence from qualitative studies will be synthesised thematically, and the results will be integrated with the quantitative evidence using the parallel synthesis or mixed methods approach [54, 55]. Recommendations for future interventions and policy decisions will be drawn by interpreting quantitative findings, using the themes identified from qualitative studies.

## Discussion

The importance of diabetes-related complications may be underestimated. Data from the 2010–2011 National Paediatric Diabetes Audit (NPDA) showed that only 5.8 % of all children and young persons with diabetes are recorded as having received all the National Institute for Health and Care Excellence (NICE) recommended care processes aimed at reducing risk of chronic complications [56]. This percentage increased to 16.1 % in the 2013–2014 NPDA, which may reflect the incentivising effect of the recent introduction of the best practice tariff for paediatric diabetes care in the UK [57]. However, this percentage of optimum service delivery still compares unfavourably to adults with diabetes (>60 % received all recommended care processes during the 2011–2012 and 2012–2013 National Diabetes Audits (NDA)) and is very low when compared internationally [16, 58].

In a clinic setting, aiming for tight glycaemic targets remains difficult to achieve, outside of a clinical trial. The mean HbA1_c_ level in the intensively treated group participating for more than 20 years ago in the DCCT was lower than the HbA1_c_ levels in most patients today [59, 60]. The intensive group achieved HbA1_c_ levels of 7 % on an average compared with 8.3 % among more than 25,000 patients from 67 US centres in the T1D exchange [59], and this was achieved without modern advances in therapy, such as insulin analogues and continuous glucose monitors. Therefore, we need to understand the effect, predictors and trends of early glycaemic control on complications risk in childhood and adults.

Our second aim is to look at which factors predict early HbA1_c_. These could be factors across the whole biopsychosocial model. For example, there may be social factors (deprivation, ethnic), psychological factors (teenage behaviour, fear of hypoglycaemia), physiological factors (glycation index) as well as wider cultural factors (the family, school, clinic setting, etc.).

By systematically reviewing evidence on effect of glycaemic control within first 2 years of T1D diagnosis and tracking with increasing diabetes duration, this will help identify predictors of early glycaemic control and understand future risk for complications in the paediatric population.

### How this review compares with previous reviews in children and young adults

To our knowledge, this is the first review to robustly investigate evidence on the association of early glycaemic control in childhood-onset T1D with future complications risk. Furthermore, this is the first review to rigorously and systematically integrate quantitative (both intervention and observational) and qualitative evidence on this topic [55]. Evidence synthesised this way is holistic and more reliable than syntheses of any one type of research in isolation.

### Dissemination and updating plans

Results of the review will be disseminated through peer-reviewed publications, conference presentations and at meetings. The review will be updated if significant new evidence becomes available.

## Additional file

Additional file 1:
**Electronic database search strategy.**

## Abbreviations

DCCT: Diabetes Control and Complications Trial
EPPI: Evidence for Policy and Practice Information
HbA1_c_: glycated haemoglobin A1_c_
NDA: National Diabetes Audit
NICE: National Institute for Health and Care Excellence
NPDA: National Paediatric Diabetes Audit
PROSPERO: International Prospective Register for systematic Reviews
RCT: randomised control trial
T1D: type 1 diabetes
