# Supplementary material for: Protocol for systematic review of evidence on the determinants and influence of early glycaemic control in childhood-onset type 1 diabetes
Source: Syst Rev. 2015 Nov 12;4:159. doi: 10.1186/s13643-015-0146-8 (PMC4643492; doi:10.1186/s13643-015-0146-8)
Supplement: Additional file 1: — Electronic database search strategy. [file 13643_2015_146_MOESM1_ESM.pdf]

Additional file 1: Electronic database search strategy

I.	**Scopus (via Elsevier) (17/12/2014)**
1.	(TITLE-ABS-KEY (({early intensive} OR tight OR glycemic OR glycaemic OR glucose OR diabetes OR strict) W/2 control) OR TITLE-ABS-KEY (insulin W/2 (use* OR injection* OR dose* OR pump*)) OR TITLE-ABS-KEY (glycosylat* OR {HbA1c} OR a1c OR hemoglobin a OR haemoglobin OR {HbA(1c)}) OR TITLE-ABS-KEY ((intensive OR conventional OR standard OR regular OR optimised OR usual OR routine) W/2 (care OR treatment OR therapy OR intervention OR management)) OR TITLE-ABS-KEY (hyperglycaemia OR hypoglycaemia)) AND (TITLE-ABS-KEY OR ({Diabetes complication*} OR {side effects} OR {adverse events} OR glycemia OR glycaemia OR {hyper glycemia} OR {hyper glycaemia} OR hyperglycemia OR hyperglycaemia OR {hypo glycemia} OR {hypo glycaemia} OR hypoglycemia OR hypoglycaemia) OR TITLE-ABS-KEY (ketosis OR {diabetic ketoacidosis} OR dka OR {nonketotic hyperosmolar coma} OR {insulin resistance} OR {autoimmune disease*} OR {auto immune disease}) OR TITLE-ABS-KEY ({urine albumin} OR microalbuminaria OR macroalbuminuria OR {renal disease*} OR {kidney disease*} OR {diabetic nephropathy} OR nephropathy OR dialysis) OR TITLE-ABS-KEY ({foot ulcer} OR amputation) OR TITLE-ABS-KEY (retinopathy OR blindness OR {cardiovascular disease*} OR mi OR {myocardial infarction*} OR stroke* OR {coronary artery disease*}) OR TITLE-ABS-KEY ({cerebrovascular disease*} OR {peripheral vascular disease*} OR {blood pressure} OR bp OR statin* OR death OR mortality)) AND (TITLE-ABS-KEY (pediatric OR paediatric OR child* OR {young people} OR youth OR {young adult*} OR juvenile OR {insulin dependent} OR labile OR brittle OR {sudden onset} OR autoimmune OR {auto immune} OR {non insulin dependent} OR uncontrolled OR {newly diagnosed} OR {new diagnosis} OR {inception diabetes})) AND (TITLE-ABS-KEY (dm1 OR {diabetes mellitus 1} OR {diabetes mellitus} W/2 {type 1}) OR t1d OR t1dm OR iddmor {type 1}) AND TITLE-ABS-KEY (metabolism OR {metabolic memory})
2	TITLE-ABS-KEY (metabolism OR {metabolic memory})
3	(TITLE-ABS-KEY or ({Diabetes complication*} or {side effects} or {adverse events} or glycemia or glycaemia or {hyper glycemia} or {hyper glycaemia} or hyperglycemia or hyperglycaemia or {hypo glycemia} or {hypo glycaemia} or hypoglycemia or hypoglycaemia) or TITLE-ABS-KEY (ketosis or {diabetic ketoacidosis} or DKA or {nonketotic hyperosmolar coma} or {insulin resistance} or {autoimmune disease*} or {auto immune disease}) or TITLE-ABS-KEY ({urine albumin} or microalbuminaria or macroalbuminuria or {renal disease*} or {kidney disease*} or {diabetic nephropathy} or nephropathy or dialysis) or TITLE-ABS-KEY ({foot ulcer} or amputation) or TITLE-ABS-KEY (retinopathy or blindness or {cardiovascular disease*} or MI or {myocardial infarction*} or stroke* or {coronary artery disease*}) or TITLE-ABS-KEY ({cerebrovascular disease*} or {peripheral vascular disease*} or {blood pressure} or BP or statin* or death or mortality))
4	(TITLE-ABS-KEY (({early intensive} or tight or glycemic or glycaemic or glucose or diabetes or strict) W/2 control) OR TITLE-ABS-KEY (insulin W/2 (use* or injection* or dose* or pump*)) OR TITLE-ABS-KEY (glycosylat* or {HbA1c} or A1c or Hemoglobin A or haemoglobin

	or {HbA(1c)}) OR TITLE-ABS-KEY((intensive or conventional or standard or regular or optimised or usual or routine) W/2 (care or treatment or therapy or intervention or management)) OR TITLE-ABS-KEY (hyperglycaemia or hypoglycaemia))
5	(TITLE-ABS-KEY (pediatric OR paediatric OR child* OR {young people} OR youth OR {young adult*} OR juvenile OR {insulin dependent} OR labile OR brittle OR {sudden onset} OR autoimmune OR {auto immune} OR {non insulin dependent} OR uncontrolled OR {newly diagnosed} OR {new diagnosis} OR {inception diabetes}))
6	(TITLE-ABS-KEY (dm1 OR {diabetes mellitus 1} OR {diabetes mellitus} W/2 {type 1}) OR t1d OR t1dm OR iddmor {type 1})

**II.**	**Cochrane Library (17/12/2014)**
#1	MeSH descriptor: [Diabetes Mellitus, Type 1] explode all trees
#2	DM1 or diabetes mellitus 1 or diabetes mellitus type 1 or T1D or T1DM or IDDM
#3	type 1 or paediatric or child or young people or youth or young adults or juvenile or insulin dependent or labile or brittle or sudden onset or autoimmune or auto immune or non insulin dependent or uncontrolled or newly diagnosed or new diagnosis or inception diabetes
#4	#1 or #2 or #3
#5	MeSH descriptor: [Blood Glucose] explode all trees
#6	MeSH descriptor: [Hemoglobin A, Glycosylated] explode all trees
#7	MeSH descriptor: [Hypoglycemia] explode all trees
#8	MeSH descriptor: [Hyperglycemia] explode all trees
#9	#5 or #6 or #7 or #8
#10	early intensive or tight or glycemic or glucose or diabetes or strict control
#11	insulin use or injection or dose or pump
#12	glycosylate or HbA1c or A1c or Hemoglobin A or HbA1c

#13	intensive or conventional or standard or regular or optimised or usual or routine care or treatment or therapy or intervention or management
#14	#9 or #10 or #11 or #12 or #13
#15	MeSH descriptor: [Diabetes Complications] explode all trees
#16	adverse effects or complications
#17	MeSH descriptor: [Ketosis] explode all trees
#18	MeSH descriptor: [Insulin Resistance] explode all trees
#19	MeSH descriptor: [Autoimmune Diseases] explode all trees
#20	MeSH descriptor: [Albuminuria] explode all trees
#21	MeSH descriptor: [Kidney Diseases] explode all trees
#22	MeSH descriptor: [Dialysis] explode all trees
#23	MeSH descriptor: [Blindness] explode all trees
#24	MeSH descriptor: [Cardiovascular Diseases] explode all trees
#25	MeSH descriptor: [Cerebrovascular Disorders] explode all trees
#26	MeSH descriptor: [Blood Pressure] explode all trees
#27	MeSH descriptor: [Hydroxymethylglutaryl-CoA Reductase Inhibitors] explode all trees
#28	MeSH descriptor: [Mortality] explode all trees
#29	Diabetes complications or side effects or adverse events or glycaemia or hyper glycaemia or hypo glycaemia or ketosis or diabetic ketoacidosis or DKA or nonketotic hyperosmolar coma or insulin resistance or autoimmune disease or urine albumin or urine albumin creatinine ratio or urine albumin excretion or microalbuminuria or macroalbuminuria or renal disease or diabetic nephropathy or nephropathy or dialysis or foot ulcer or amputation or retinopathy or blindness or cardiovascular disease or MI or myocardial infarction or

	stroke or coronary artery disease or cerebrovascular disease or peripheral vascular disease or blood pressure or BP or statin or death or mortality
#30	#15 or #16 or #17 or #18 or #19 or #20 or #21 or #22 or #23 or #24 or #25 or #26 or #27 or #28 or #29
#31	metabolism
#32	metabolic memory
#33	#31 or #32
#34	#4 and #9 and #14 and #30 and #33

**III.**	**CINAHL (via EBSCO) (16/12/2014)**
S34	S4 AND S14 AND S30 AND S33
S33	S31 OR S32
S32	"metabolic memory"
S31	MJ metabolism
S30	S15 OR S16 OR S17 OR S18 OR S19 OR S20 OR S21 OR S22 OR S23 OR S24 OR S25 OR S26 OR S27 OR S28 OR S29
S29	diabetes complication or diabetes complication* or side effects or adverse events or glyc#emia or hyper glyc#emia or hyperglyc#emia or hypo glyc#emia or hypoglyc#emia or ketosis or diabetic ketoacidosis or DKA or nonketotic hyperosmolar coma or insulin resistance or autoimmune disease* or urine albumin or microalbuminaria or macroalbuminuria or renal disease* or kidney disease* or diabetic nephropathy or nephropathy or dialysis or foot ulcer or amputation or retinopathy or blindness or cardiovascular disease* or MI or myocardial infarction* or stroke* or coronary artery disease* or cerebrovascular disease* or peripheral vascular disease* or blood pressure or BP or statin* or death or mortality
S28	(MH "mortality+")
S27	(MH "statins+")
S26	(MH "blood pressure+")
S25	(MH "cerebrovascular disorders+")
S24	(MH "stroke+")

S23	(MH "cardiovascular diseases+")
S22	(MH "blindness+")
S21	(MH "dialysis+")
S20	(MH "kidney diseases+")
S19	(MH "Albuminuria")
S18	(MH autoimmune diseases+)
S17	(MH insulin resistance+)
S16	(MH "diabetic angiopathies+") OR (MH "diabetic cardiomyopathies") OR (MH "diabetic coma+") OR (MH "diabetic ketoacidosis") OR (MH "diabetic neuropathies+")
S15	(MH "diabetes mellitus/co")
S14	S5 OR S6 OR S7 OR S8 OR S9 OR S10 OR S11 OR S12 OR S13
S13	(intensive OR conventional OR standard OR regular OR optimi#ed OR usual OR routine) N2 (care OR treatment OR therapy OR intervention OR management)
S12	glycosylat* OR HbA1c OR A1c OR H#emoglobin A OR HbA#1c
S11	insulin N2 (use* OR injection* OR dose* OR pump*)
S10	(“early intensive” OR tight OR glyc#emic OR glucose OR diabetes or strict) N2 control)
S9	(MH "Hyperglycemia+")
S8	(MH "Hypoglycemia+")
S7	MH blood glucose
S6	MH hemoglobin a, glycosylated
S5	(MH "Glycemic Control")
S4	S1 OR S2 OR S3
S3	("type 1" OR p#ediatric OR child* OR "young people" OR youth OR "young adult" OR juvenile OR "insulin dependent" OR labile OR brittle OR "sudden onset" OR autoimmune OR "auto immune" OR "non insulin dependent" OR uncontrolled OR "newly diagnosed" OR "new diagnosis" OR inception) N5 diabetes
S2	DM1 OR "diabetes mellitus 1" OR ("diabetes mellitus" N2 type 1) OR T1D or T1DM or IDDM
S1	(MH "Diabetes Mellitus, Type 1+")

**IV.**	**Web of Science (via Thomson Reuters) (16/12/2014)**
1	TOPIC: ((DM1 OR "diabetes mellitus 1" OR ("diabetes mellitus" NEAR/2 "type 1") OR T1D or T1DM or IDDM) OR TOPIC: (("type 1" OR p$ediatric OR child* OR "young people" OR youth OR "young adult" OR juvenile OR "insulin dependent" OR labile OR brittle OR "sudden

2	onset" OR autoimmune OR "auto immune" OR "non insulin dependent" OR uncontrolled OR "newly diagnosed" OR "new diagnosis" OR inception) NEAR/5 diabetes))<br>Indexes=SCI-EXPANDED, SSCI, A&HCI, CPCI-S, CPCI-SSH, BKCI-S, BKCI-SSH, CCR-EXPANDED, IC Timespan=All years
2	TOPIC: (TOPIC: ((“early intensive” OR tight OR glyc$emic OR glucose OR diabetes or strict) NEAR/2 control) OR TOPIC: (insulin NEAR/2 (use* OR injection* OR dose* OR pump*)) OR TOPIC: (glycosylat* OR HbA1c OR A1c OR H$emoglobin A OR HbA$1c) OR TOPIC: ((intensive OR conventional OR standard OR regular OR optimi$ed OR usual OR routine) NEAR/2 (care OR treatment OR therapy OR intervention OR management)) OR TOPIC: (hyperglyc$emia OR hypoglyc$emia))<br>Indexes=SCI-EXPANDED, SSCI, A&HCI, CPCI-S, CPCI-SSH, BKCI-S, BKCI-SSH, CCR-EXPANDED, IC Timespan=All years
3	TOPIC: (TOPIC: (“Diabetes complication*” OR “side effects” OR “adverse events” OR glyc$emia OR “hyper glyc$emia” OR hyperglyc$emia OR “hypo glyc$emia” OR hypoglyc$emia OR ketosis OR “diabetic ketoacidosis” OR DKA OR “nonketotic hyperosmolar coma” OR “insulin resistance” OR “autoimmune disease*” OR “auto immune disease” OR “urine albumin” OR microalbuminaria OR macroalbuminuria OR “renal disease*” OR “kidney disease*” OR nephropathy OR dialysis OR “foot ulcer” OR amputation OR retinopathy OR blindness OR “cardiovascular disease*” OR MI OR “myocardial infarction*” OR stroke* OR “coronary artery disease*” OR “cerebrovascular disease*” OR “peripheral vascular disease*” OR “blood pressure” OR BP OR statin* OR death OR mortality))<br>Indexes=SCI-EXPANDED, SSCI, A&HCI, CPCI-S, CPCI-SSH, BKCI-S, BKCI-SSH, CCR-EXPANDED, IC Timespan=All years
4	TOPIC: (TOPIC: (metabolism OR "metabolic memory" OR metabolic))<br>Indexes=SCI-EXPANDED, SSCI, A&HCI, CPCI-S, CPCI-SSH, BKCI-S, BKCI-SSH, CCR-EXPANDED, IC Timespan=All years

**V.**	**EMBASE (via OVID) (16/12/2014)**
1	exp insulin dependent diabetes mellitus/
2	(DM1 or diabetes mellitus 1 or (diabetes mellitus adj2 type 1) or T1D or T1DM or IDDM).mp. [mp=title, abstract, subject headings, heading word, drug trade name, original title, device manufacturer, drug manufacturer, device trade name, keyword]
3	((type 1 or p?ediatric or child* or young people or youth or young adults or juvenile or insulin dependent or labile or brittle or sudden onset or autoimmune or auto immune or non insulin dependent or uncontrolled or newly diagnosed or new diagnosis or inception) adj5 diabetes).mp. [mp=title, abstract, subject headings, heading word, drug trade name, original title, device manufacturer, drug manufacturer, device trade name, keyword]
4	1 or 2 or 3
5	exp glycosylated hemoglobin/
6	exp glucose blood level/
7	exp hypoglycemia/
8	exp hyperglycemia/

9	((early intensive or tight or glyc?emic or glucose or diabetes or strict) adj2 control).mp. [mp=title, abstract, subject headings, heading word, drug trade name, original title, device manufacturer, drug manufacturer, device trade name, keyword]
10	(insulin adj2 (use* or injection* or dose* or pump*)).mp. [mp=title, abstract, subject headings, heading word, drug trade name, original title, device manufacturer, drug manufacturer, device trade name, keyword]
11	(glycosylat* or HbA1c or A1c or H?emoglobin A or HbA?1c).mp. [mp=title, abstract, subject headings, heading word, drug trade name, original title, device manufacturer, drug manufacturer, device trade name, keyword]
12	((intensive or conventional or standard or regular or optimi?ed or usual or routine) adj2 (care or treatment or therapy or intervention or management)).mp. [mp=title, abstract, subject headings, heading word, drug trade name, original title, device manufacturer, drug manufacturer, device trade name, keyword]
13	5 or 6 or 7 or 8 or 9 or 10 or 11 or 12
14	diabetic angiopathy/ or diabetic cardiomyopathy/ or diabetic coma/ or diabetic foot/ or diabetic hypertension/ or diabetic ketoacidosis/ or diabetic macular edema/ or diabetic nephropathy/ or diabetic neuropathy/ or diabetic obesity/ or diabetic retinopathy/ or impaired glucose tolerance/ or "maternally inherited diabetes and deafness"/ or nonketotic diabetic coma/ or wolfram syndrome/
15	exp diabetes mellitus/co [Complication]
16	exp diabetes mellitus/si [Side Effect]
17	exp insulin resistance/
18	exp autoimmune disease/
19	exp albuminuria/
20	exp kidney disease/
21	exp dialysis/
22	exp blindness/
23	exp cardiovascular disease/
24	exp cerebrovascular disease/
25	exp blood pressure/
26	exp hydroxymethylglutaryl coenzyme A reductase inhibitor/
27	exp mortality/
28	(Diabetes complication* or side effects or adverse events or glyc?emia or hyper glyc?emia or hyperglyc?emia or hypo glyc?emia or hypoglyc?emia or ketosis or diabetic ketoacidosis or DKA or nonketotic hyperosmolar coma or insulin resistance or autoimmune disease* or urine albumin or microalbuminaria or macroalbuminuria or renal disease* or kidney disease* or diabetic nephropathy or nephropathy or dialysis or foot ulcer or amputation or retinopathy or blindness or cardiovascular disease* or MI or myocardial infarction* or stroke* or coronary artery disease* or cerebrovascular disease* or peripheral vascular disease* or blood pressure or BP or statin* or death or mortality).mp. [mp=title, abstract, subject headings, heading word, drug trade name, original title, device manufacturer, drug manufacturer, device trade name, keyword]
29	14 or 15 or 16 or 17 or 18 or 19 or 20 or 21 or 22 or 23 or 24 or 25 or 26 or 27 or 28

30	memory/
31	metabolic memory.mp. [mp=title, abstract, subject headings, heading word, drug trade name, original title, device manufacturer, drug manufacturer, device trade name, keyword]
32	30 or 31
33	4 and 13 and 29 and 32

VI.	Medline (via OVID) (16/12/2014)
1	exp Diabetes Mellitus, Type 1/
2	(((DM1 or diabetes mellitus 1 or diabetes mellitus) adj2 type 1) or T1D or T1DM or IDDM).mp. [mp=title, abstract, original title, name of substance word, subject heading word, keyword heading word, protocol supplementary concept word, rare disease supplementary concept word, unique identifier]
3	((type 1 or p?ediatric or child* or young people or youth or young adults or juvenile or insulin dependent or labile or brittle or sudden onset or autoimmune or auto immune or non insulin dependent or uncontrolled or newly diagnosed or new diagnosis or inception) adj5 diabetes).mp. [mp=title, abstract, original title, name of substance word, subject heading word, keyword heading word, protocol supplementary concept word, rare disease supplementary concept word, unique identifier]
4	1 or 2 or 3
5	exp Blood Glucose/ or exp Hemoglobin A, Glycosylated/ or exp Hypoglycemia/
6	exp Hyperglycemia/
7	((early intensive or tight or glyc?emic or glucose or diabetes or strict) adj2 control).mp. [mp=title, abstract, original title, name of substance word, subject heading word, keyword heading word, protocol supplementary concept word, rare disease supplementary concept word, unique identifier]
8	(insulin adj2 (use* or injection* or dose* or pump*)).mp. [mp=title, abstract, original title, name of substance word, subject heading word, keyword heading word, protocol supplementary concept word, rare disease supplementary concept word, unique identifier]
9	(glycosylat* or HbA1c or A1c or H?emoglobin A or HbA?1c).mp. [mp=title, abstract, original title, name of substance word, subject heading word, keyword heading word, protocol supplementary concept word, rare disease supplementary concept word, unique identifier]
10	((intensive or conventional or standard or regular or optimi?ed or usual or routine) adj2 (care or treatment or therapy or intervention or management)).mp. [mp=title, abstract, original title, name of substance word, subject heading word, keyword heading word, protocol supplementary concept word, rare disease supplementary concept word, unique identifier]
11	5 or 6 or 7 or 8 or 9 or 10
12	exp Diabetes Complications/
13	adverse effects.fs.
14	complications.fs.

15	exp Ketosis/
16	exp Insulin Resistance/
17	exp Autoimmune Diseases/
18	exp Albuminuria/
19	exp Kidney Diseases/
20	exp Dialysis/
21	exp Blindness/
22	exp Cardiovascular Diseases/
23	exp Cerebrovascular Disorders/
24	exp Blood Pressure/
25	exp Hydroxymethylglutaryl-CoA Reductase Inhibitors/
26	exp Mortality/
27	(Diabetes complications or side effects or adverse events or glycaemia or hyper glycaemia or hypo glycaemia or ketosis or diabetic ketoacidosis or DKA or nonketotic hyperosmolar coma or insulin resistance or autoimmune disease or urine albumin or urine albumin creatinine ratio or urine albumin excretion or microalbuminuria or macroalbuminuria or renal disease or diabetic nephropathy or nephropathy or dialysis or foot ulcer or amputation or retinopathy or blindness or cardiovascular disease or MI or myocardial infarction or stroke or coronary artery disease or cerebrovascular disease or peripheral vascular disease or blood pressure or BP or statin or death or mortality).mp. [mp=title, abstract, original title, name of substance word, subject heading word, keyword heading word, protocol supplementary concept word, rare disease supplementary concept word, unique identifier]
28	12 or 13 or 14 or 15 or 16 or 17 or 18 or 19 or 20 or 21 or 22 or 23 or 24 or 25 or 26 or 27
29	4 and 11 and 28
30	metabolism.fs.
31	metabolic memory.mp. [mp=title, abstract, original title, name of substance word, subject heading word, keyword heading word, protocol supplementary concept word, rare disease supplementary concept word, unique identifier]
32	30 or 31
33	29 and 32
